# Ultrathin Ni-MOF Nanobelts-Derived Composite for High Sensitive Detection of Nitrite

**DOI:** 10.3389/fchem.2020.00330

**Published:** 2020-04-23

**Authors:** Xiangren Meng, Xiao Xiao, Huan Pang

**Affiliations:** ^1^School of Tourism and Culinary Science, Yangzhou University, Yangzhou, China; ^2^Jiangsu Huai-yang Cuisine Engineering Center, Yangzhou University, Yangzhou, China; ^3^School of Chemistry and Chemical Engineering, Yangzhou University, Yangzhou, China

**Keywords:** in suit conversion method, Ni/NiO ultrathin nanobelt, metal nanoparticle, metal organic framework, nitrite

## Abstract

In this paper, the Ni/NiO ultrathin nanobelts were successively synthesized by a facile in suit conversion process using pre-synthesized Ni-based metal-organic frameworks (MOFs) nanobelts as parent materials to detect the nitrite (NaNO_2_). The synthesized Ni/NiO composites have the advantages in structure, as follows: (I) Interleaved 3D reticulated structure has strong mechanical stability; (II) Ultrathin nanobelt structures allow more active sites to be exposed and make the transfer of charge faster; (III) A large number of ultrafine Ni nanoparticles decorate the building blocks of the NiO nanobelt and enhance the electrical conductivity. Ni/NiO/GCE has an obvious oxidation peak at 0.78 V, when the concentration is between 0.5 and 1000 μM, the oxidation peak current of NaNO_2_ is linearly related to the concentration, and the sensitivity is 1.5319 μA mM^−1^ cm^−2^ (S/N = 3). Moreover, the experimental results also concluded that the Ni/NiO ultrathin nanobelts not only indicated wonderful reproducibility in the determination of NaNO_2_ in the pickled pork samples, but also could be well-recovered and keep stable for a long time.

## Introduction

Metal-organic frameworks (MOFs) are expected to play a significant role in energy applications (Lin et al., 2015; Xia et al., 2015; Tan et al., 2017; Liang et al., 2018; Wu et al., 2019; Yang et al., 2019; Zhao Y. et al., 2019). Because the micro/nanoscale structure has a great potential to overcome the disadvantages of low specific surface area (SSAs) and poor contacts of active materials with electrolyte/pollutants compared to conventional bulks or aggregate materials. Therefore, it is considered as a promising electrode material (Du et al., 2014, 2017; Bosch et al., 2017; Chen et al., 2017; Shi et al., 2017; Li Y.-P. et al., 2019; Xiao et al., 2020). In recent years, since it has been noted that various processes such as thermodynamics and kinetics of various reactions occurring at the interface are significantly affected by the surface energy of micro/nanocrystals, MOF-derived materials have become a research hotspot (Yang et al., 2008; Larsson et al., 2009; Du et al., 2014, 2017; Bosch et al., 2017; Chen et al., 2017; Shi et al., 2017; Liu et al., 2019; Li Y.-P. et al., 2019; Xiao et al., 2020). Many studies have focused on designing and controlling their different morphologies. On the basis of keeping the original geometry of the micro/nanostructure unchanged, the derivatization can improve the low conductivity and enhance the structural stability of the original MOFs (Chen et al., 2012; Liu et al., 2014; Ma et al., 2017; Shi et al., 2017; Wang et al., 2018; Zou et al., 2018).

Because of its great importance in catalysis, metal nanoparticles are rapidly attracting widespread interest (Han et al., 2010; Zhang et al., 2010, 2012, 2014; Xu et al., 2014; Li Q. Y. et al., 2019; Zhu et al., 2019). However, the high surface energy of metal nanoparticles makes them thermodynamically unstable during the process of catalytic reaction, and it is easy to aggregate, resulting in reduced activity. Therefore, whether the size, shape and dispersion of metal nanoparticles can be controlled properly is the key factor to determine whether the stability activity can be improved. To the end, small metal nanoparticles with specific shapes are generally prepared from many surface capping agents including dendrimers, oleamide, and polyethylenepyrrolidone (PVP) (Cho et al., 2016; Zhao et al., 2016; Li et al., 2018; Zhang et al., 2019; Zhao R. B. et al., 2019). Although this surface capping agent is considered effective, in most cases, it is not ideal to attach molecules to metal nanoparticles with strong chemical interactions and inhibit the catalytic reaction. In order to produce surface-clean and well-dispersed metal nanoparticles, limiting them within porous materials, such as porous silica, zeolites, as well as porous carbons are currently common methods. Through the porous materials, the pore-responsive substrates/products can be transferred, porous materials can also avoid the aggregation and growth of metal nanoparticles, all of which are attributed to its inherent conditions for spatial confinement (Zhan and Zeng, 2016, 2018; Chen and Xu, 2017; Xu et al., 2017; Li et al., 2018; Geng et al., 2019). MOFs and MOF-derived materials can stand out among numerous porous materials and become excellent choices because (1) they have diverse pore sizes and shapes to meet the special requirements of metal nanoparticles; (2) They have high porosity and specific surface areas to carry metal nanoparticles; (3) Understanding catalysis requires defining the MOF structure distinctly and ensuring that the pore structure is easily tailored in order to assure that the surrounding environment of metal nanoparticles is easily identified (Mukoyoshi et al., 2015; Tang et al., 2018; Chen et al., 2019; Li Y.-P. et al., 2019).

Nitrite, mainly sodium nitrite (NaNO_2_), is often used as a preservative and a food additive in daily life. The high content of nitrite in the human body not only causes hemoglobin to be irreversibly oxidized to high-iron hemoglobin, but also reacts with dietary components to produce nitrosamines, leading to cancer and high blood pressure (Yue et al., 2011; Lin Z. et al., 2015). Therefore, eating meat products, sauces and spoiled vegetables with higher levels of nitrate or nitrite, or the drinking water containing nitrate or nitrite can cause poisoning (Li et al., 2012; Zou et al., 2017). In this work, we report that, with pre-synthesized ultrathin Ni-MIL-77 nanobelts as parent materials, a facile *in situ* conversion way (O_2_-protected annealing process) has been demonstrated to fabricate metal nickel nanoparticle functionalized NiO composites (Ni/NiO ultrathin nanobelts). An excellent electrochemical performance of the Ni/NiO ultrathin nanobelts with large surface area and metal Ni nanoparticles generated by *in situ* conversion method is exhibited during the oxidation of NaNO_2_. In addition, NaNO_2_ in pickled pork was determined by the Ni/NiO materials, and good recovery was achieved.

## Results and Discussion

### Synthesis Strategy

**Figure 1:**
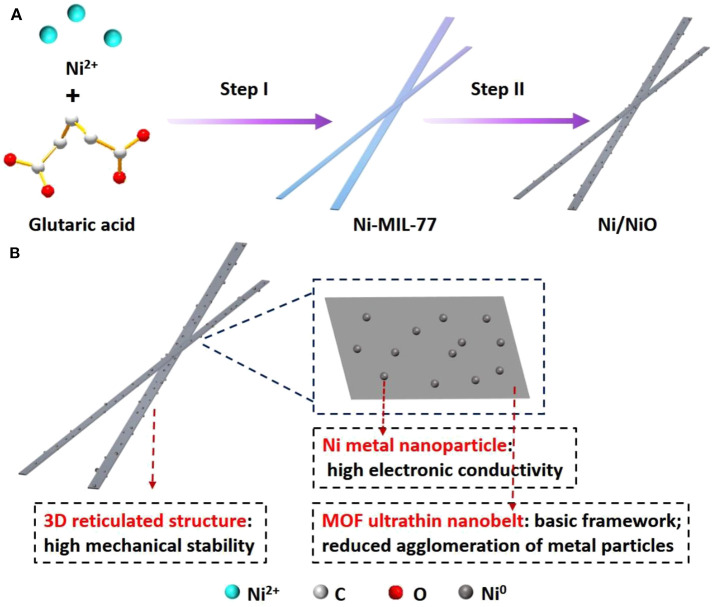
**(A)** Schematic illustration of the synthesis of Ni/NiO ultrathin nanobelts. Step I: hydrothermal treatment of Ni-MIL-77 ultrathin nanobelts; Step II: annealing the Ni-MOF ultrathin nanobelts in air. **(B)** Schematic illustration of the detailed Ni/NiO ultrathin nanobelts.

In this work, we used Ni-MIL-77 as the precursor because it presented a chiral structure that has large cross channels (Figure S1). The Ni/NiO composite reported here was made by using an in suit conversion process. Two steps are included in the preparation of Ni-functionalized NiO ultrathin nanobelts (Figure 1A). In step I, we successfully synthesized Ni-MIL-77 ultrathin nanobelts under solvothermal conditions (Xiao et al., 2017, 2018, 2019). In step II, we used a method that anneals the nickel-based ultrathin nanobelts in air at an elevated temperature to make it converted into Ni/NiO heterostructure nanobelts *in situ*. There are many structural features of the obtained Ni/NiO composite (Figure 1B): (I) interleaved 3D reticulated structure has strong mechanical stabilities; (II) More active sites are exposed and the charge transfer process is also promoted due to ultrathin nanobelt structures; (III) A large number of ultrafine Ni nanoparticles decorate the building blocks of the NiO nanobelt and enhance the electrical conductivity.

### Characterizations of Ni/NiO Heterostructure

The thermogravimetric (TG) curve of Ni-MIL-77 that shows two obvious weight losses is given in Figure S2. In the temperature range of 25 to100°C is the first step of the weight loss (a 6% drop), which is equivalent to the decrease of the adsorbed solvent molecules. While the temperature reaches 400°C, since the Ni-MIL-77 skeleton is decomposed and glutaric acid (HOOC(CH_2_)_3_COOH) ligands turn into gas, weight loses 40.1%. Therefore, we can obtain Ni/NiO heterostructures at 350°C.

**Figure 2:**
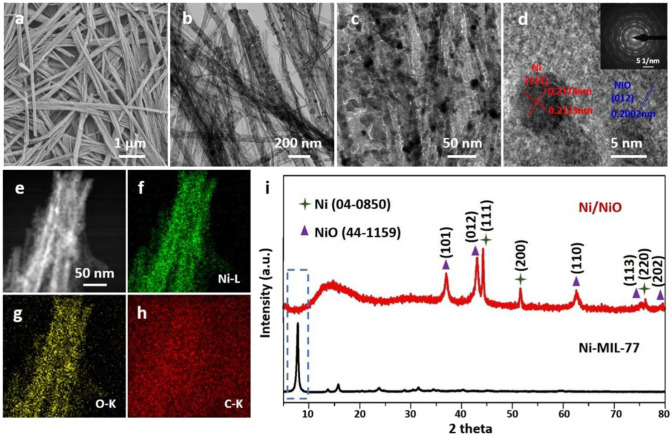
Morphology and structure characterization of Ni/NiO ultrathin nanobelts. **(a)** SEM, **(b,c)** TEM, **(d)** HRTEM (insert: SAED patterns) and **(e–h)** elemental mapping images of Ni/NiO ultrathin nanobelts. **(i)** XRD patterns of the Ni-MIL-77 and Ni/NiO.

The morphology of the samples can be observed using transmission electron microscopy (TEM) and field emission scanning electron microscopy (FESEM). The typical low-magnification SEM images in Figure S3 and TEM images in Figure S4 show that the morphology of Ni-MOF and Ni/NiO composites are ultrathin nanobelts. The results show that the thickness of Ni/NiO nanobelts is about 3 nm (Figure S5). Further understanding of the architecture of the Ni/NiO composites is shown in Figure 2. It can be clearly seen from Figures 2a,b that the Ni metal nanoparticles have been *in situ* generate. These small metal particles are uniformly distributed on the NiO-based surface, forming a stable Ni/NiO composite (Figure 2c). The rational elemental constituents (e.g., Ni, and O) of the product is shown in the energy-dispersive X-ray spectrum (EDX)-mapping without impurities in Ni/NiO ultrathin nanobelts (Figures 2d–g).

We used XRD to test the phase purity as well as the crystallographic structure of the experimental product. By calcining Ni-MIL-77 at 350°C, we found two kinds of diffraction peaks (Ni and NiO) in the Figure 2i and Figure S6. Five well-defined diffraction peaks at 37.248, 43.286, 62.852, 75.404, and 79.372 are assigned to the (101), (012), (110), (113), and (202) facets of NiO, respectively. And three well-defined diffraction peaks at 44.507, 51.846, and 76.370 are distributed to the (111), (200), and (220) facets of Ni, respectively. The diffraction peaks can be easily assigned to the standard NiO phase (JCPDS 44-1159) as well as Ni phase (JCPDS 04-0850) (Tang et al., 2018; Zhao L. et al., 2019).

**Figure 3:**
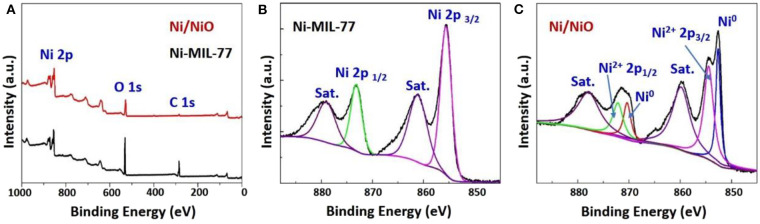
**(A)** XPS survey of the Ni-MOF and Ni/NiO; Ni 2p_3/2_ XPS spectra of **(B)** Ni-MOF **(C)** and Ni/NiO.

An effective method that can distinguish functional groups in the products is X-ray photoelectron spectroscopy (XPS). Figure 3 shows the XPS spectra of Ni-MOF nanobelts and Ni/NiO nanobelts and their survey spectra. The survey spectra (Figure 3A) show three apparent peaks at 282.63 (C 1s), 530.29 (O 1s), and 856.37 (Ni 2p), respectively, which implied that Ni-MIL-77 nanobelts and Ni/NiO nanobelts were successfully formed. Ni oxidation states are determined through performing Ni 2p XPS spectrum. Two peaks in the Ni 2p region of the XPS spectra for Ni-MIL-77 that centered at 856.1 (2p_3/2_) and 873.8 (2p_1/2_) eV corresponded to the Ni^2+^ ions in Ni-MIL-77 (Figure 3B). In addition to these peaks, a new pair of spin-orbit splitting peaks is generated at 852.8 (2p_3/2_) and 870.0 eV (2p_1/2_) (Figure 3C), corresponding to the formation of Ni nanoparticles, and the results are consistent with PXRD. Figures S7a, S8a shows the high-resolution XPS spectrum of C 1s, and it can be deconvoluted well into two surface carbon components at ≈284.3 eV (nonoxygenated carbon: C-C), as well as 288.1 eV (carboxyl carbon: O=C-O). Figures S7b, S8b shows the high-resolution XPS spectrum of O 1s.

### Determination of NaNO_2_ on the Ni/NiO/GCE

Figure S9 showed the cyclic voltammograms (CVs) of different electrodes (Ni-MOF/GCE, Ni/NiO/GCE) in 5.0 mM K_3_Fe(CN)_6_ containing 1 M KCl solution at a scan rate of 50 mV s^−1^. As displayed in Figure S9, the Ni/NiO /GCE exhibited an increase in the anodic peak current (192.66 μA) compared to Ni-MIL-77/GCE (108.23 μA).

**Figure 4:**
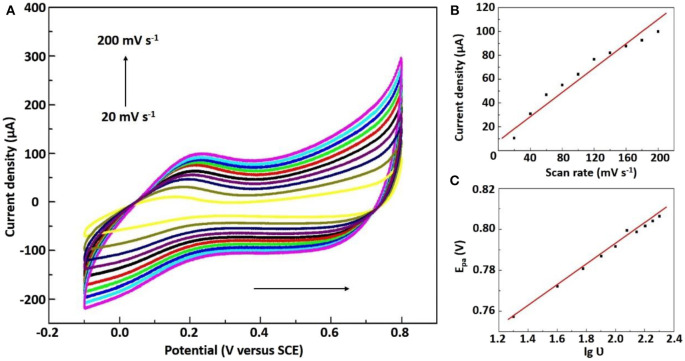
**(A)** CVs of the Ni/NiO/GCE in 0.1 M PBS solution containing 5 mM NaNO_2_ (20 to 200 mV s^−1^). **(B)** The plots of scan rates to the anodic peak currents. **(C)** lg ν vs. anodic peak potentials.

We know that studying the effect of scanning rate on the peak current and oxidation peak potential can be used to judge electrode reaction kinetics. Figure 4 and Figure S10 represented at different scan rates (20–200 mV s^−1^), the CVs of Ni/NiO/GCE and Ni-MIL-77/GCE in 0.1 M PBS solution with 5 mM NaNO_2_, respectively. The anodic peak currents for Ni/NiO/GCE increase linearly with the scan rate as well as the calibration equation is I_pa_ (μA) = 0.046465υ (mV s^−1^) + 1.35653 (*R*^2^ = 0.948) (Figure 4B). This property indicates that the electron transfer for NaNO_2_ at Ni/NiO/GCE is controlled by an adsorption process. Moreover, it can be seen that the redox peak potential changes slightly with the increase of scan rate, and the linear regression equation of E_pa_ and log of scan rate in Figure 4C is represented by E_pa_ = 0.04951lg ν + 0.69305 (*R*^2^ = 0.993). The electron transfer number (n) is calculated according to the Laviron's equation (Yang et al., 2014, 2015). It was found by calculation that there are 2e^−^ involved in the irreversible reaction of NaNO_2_, and it is consistent with Equation.

$$\begin{array}{l} NO_{2}^{-}+H_{2}O\to NO_{3}^{-}+2H^{+}+2e^{-} \end{array}$$

Figures S11, S12 describe the effect of pH on the response of 5 mM NaNO_2_ CVs with a pH range of 4.0–8.0 (scanning rate of 50 mV s^−1^). As seen from Figure S11a, the peak anode current reaches its maximum at pH 7.0. As reported by Brylev et al. (2007) N_2_O generation may lead to a decrease in peak current at low pH values. In addition, the oxidation peak of NaNO_2_ shifted in a very negative direction as pH increases, so we chose pH 7.0 as the optimal pH for our experiment.

**Figure 5:**
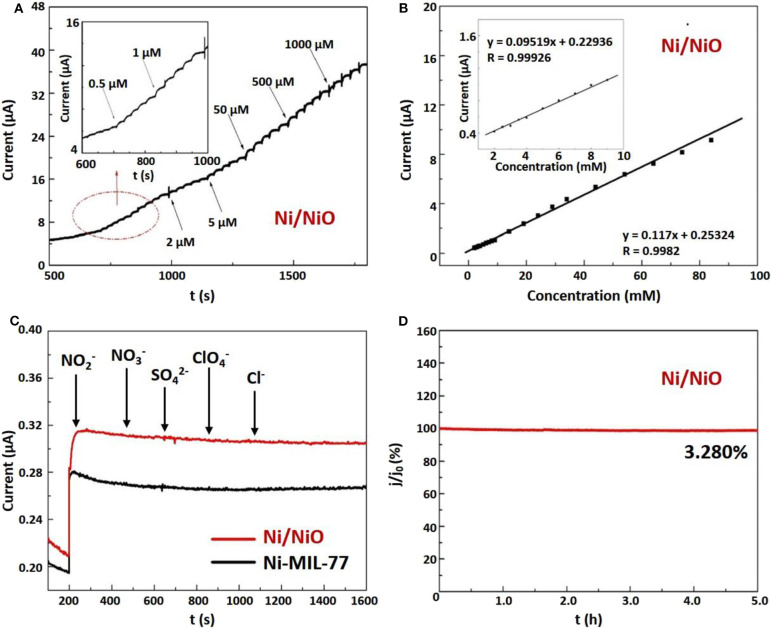
Electrochemical performances of the Ni/NiO ultrathin nanobelts modified electrode. **(A)** Amperometric responses of the Ni/NiO/GCE electrode to the successive injection of NaNO_2_ in 0.1 M PBS (inset: carefully observe the response currents of several micromolar NaNO_2_). **(B)** The calibration curve of current vs. NaNO_2_ concentration of 2~100 mM. Inset: calibration curve with NaNO_2_ concentration of 2~10 mM. **(C)** Amperometric response of Ni/NiO /GCE to ${\text{NO}}_{2}^{-}$ in the presence of ${\text{NO}}_{2}^{-}$, ${\text{NO}}_{3}^{-}$, ${\text{SO}}_{4}^{-}$, ${\text{ClO}}_{4}^{-}$, Cl^−^. **(D)** Stability of Ni/NiO/GCE over 5 h.

The linear working range of Ni/NiO/GCE was determined by recording the amperometric response as a function of NaNO_2_ concentration. Figure 5A shows the typical current time curve of the modified Ni/NiO nanobelt electrode and NaNO_2_ solution with continuous injection concentrations from 0.5 μM to 1,000 μM. An obvious reaction occurs when NaNO_2_ solution concentration is as low as 0.5 μM. Figure 5B exhibits that Ni/NiO/GCE electrode displays a sensitive increase in current response after successive increments of NaNO_2_ in 0.1 M PBS (pH = 7) solution. After each increase of NaNO_2_, the current response increases sensitively and rapidly, which shows a nice linear dependence. Two linear working ranges can be seen from Figure 5B. Notably, from the plots of electrocatalytic current versus NaNO_2_ concentration in the range of 2–100 mM (Figure 5B), the simulated linear equation of the Ni/NiO/GCE is found to be: I (*mA*) = 0.117C (*mM*) + 0.25324, R = 0.9982, and the calculated sensitivity is 1.5420 mA mM^−1^ cm^−2^. The illustrations in Figure 5B shows a good linear response in the range of 2.0~10.0 mM NaNO_2_ with a relation coefficient of 0.99926.

In the complex biological environments, the ability to identify target molecules and interference molecules is very important for sensors. Therefore, we further investigated the effects of some electroactive substances on the Ni/NiO/GCE electrode response. Figure 5C shows the amperometric response of the sensor to the consecutive addition of NaNO_2_, KNO_3_, NaSO_4_, NaClO_4_, and KCl to the solution. Only the current response of NaNO_2_ is remarkable. These results show that GCE modified by Ni/NiO ultrathin nanoribends can be used for selective and sensitive detection of NaNO_2_ without interference from KNO_3_, NaSO_4_, NaClO_4_ and KCl.

In addition, the stability of the Ni/NiO/GCE electrode was tested. The amperometric response to determining stability is shown in Figure 5D. The result exhibits that 96.72% of the current response remained unchanged for a long period of 5 h, indicating good stability in the measurement process. As shown in Figure S13, Ni-MIL-77 also exhibits good stability over a long period of 5 h, and a current response of 96.51% remains unchanged. In order to further appraise the properties of the Ni/NiO/GCE, a correlative reference is shown in Table S1.

The advantages of Ni/NiO composites can be summarized as follows:

1. The study shows that the standard electrode potential decreases ~100 mV when the electrode material size is 1 nm. Therefore, the ultrathin nanobelt structure not only allows more active sites to be exposed, but also promotes charge transfer.
2. MOF derivative support can effectively avoid agglomeration of ultrafine Ni nanoparticles, and ultrafine Ni nanoparticles can effectively improve the conductivity of MOF materials.
3. Interleaved 3D reticulated structure has strong mechanical stability.

### Determination of NaNO_2_ in Real Samples

To illustrate the feasibility and application potential of the electrode, the Ni/NiO/GCE was applied to determine NaNO_2_ from pickled pork using the standard addition technique. The collected pickled pork was disposed and the spiked NaNO_2_ concentrations were 10, 20, and 30 mg kg^−1^, respectively. As shown in Table 1, the recovery rates were 98.3, 100.3, and 99.5%, respectively. The results show that the method has good recovery rates and good practical value.

**Table 1:** Determination of various concentrations of nitrite in pickled pork.

**Samples**	**Content (mg kg^**−1**^)**	**Added (mg kg^**−1**^)**	**Found (mg kg^**−1**^)**	**Recovery (%)**	**R.S.D (%, n=10)**
1	10.2	15	24.8	98.3	2.8
2	10.2	25	35.3	100.3	2.6
3	10.2	35	45.0	99.5	3.2

## Conclusions

In summary, Ni/NiO ultrathin nanobelts were prepared by a facile in suit conversion method (O_2_-protected annealing process). The ultrathin nanobelts with large surface area and *in situ* generated metal Ni particles exhibit outstanding electrochemical performance. During the oxidation of NaNO_2_, the Ni/NiO ultrathin nanobelts exhibit excellent stability, special reproducibility, and strong anti-interference ability. Moreover, Ni/NiO composites also obtained a good recovery rate when it was applied to the determination of NaNO_2_ in marinated pork. Compared with other methods, the electrochemical method is inexpensive, simple, which is suitable for practical applications.

## Data Availability Statement

All datasets generated for this study are included in the article/Supplementary Material.

## Author Contributions

XM and XX conducted all the major experiments, designed the study, and wrote the manuscript. HP provided valuable inputs for the study's development and helped with manuscript writing. All authors agree to be accountable for the content of the work.

## Conflict of Interest

The authors declare that the research was conducted in the absence of any commercial or financial relationships that could be construed as a potential conflict of interest. The reviewer GZ declared a past co-authorship with one of the authors HP to the handling editor.
