# Corrigendum: Ultrathin Ni-MOF Nanobelts-Derived Composite for High Sensitive Detection of Nitrite

**DOI:** 10.3389/fchem.2020.00631

**Published:** 2020-07-28

**Authors:** Xiangren Meng, Xiao Xiao, Huan Pang

**Affiliations:** ^1^School of Tourism and Culinary Science, Yangzhou University, Yangzhou, China; ^2^Jiangsu Huai-yang Cuisine Engineering Center, Yangzhou University, Yangzhou, China; ^3^School of Chemistry and Chemical Engineering, Yangzhou University, Yangzhou, China

**Keywords:** in suit conversion method, Ni/NiO ultrathin nanobelt, metal nanoparticle, metal organic framework, nitrite

In the original article, there was a mistake in Figure S9 as published. When processing the CV curves, we misarranged the order of Ni/NiO and Ni-MIL-77 in Figure S9, resulting in errors.

Figure S9 shows the cyclic voltammograms (CVs) of different electrodes (Ni-MOF/GCE, Ni/NiO/GCE) in 5.0 mM K_3_Fe(CN)_6_ containing 1 M KCl solution at a scan rate of 50 mV s^−1^. As displayed in Figure S9, the Ni/NiO /GCE exhibited an increase in the anodic peak current (117.64 μA) compared to Ni-MIL-77/GCE (68.96 μA).

The authors apologize for this error and state that this does not change the scientific conclusions of the article in any way. The original article has been updated.

