# Supplementary material for: Ultrathin Ni-MOF Nanobelts-Derived Composite for High Sensitive Detection of Nitrite
Source: Front Chem. 2020 Apr 23;8:330. doi: 10.3389/fchem.2020.00330 (PMC7192062; doi:10.3389/fchem.2020.00330)
Supplement: Supplementary file 1 [file Data_Sheet_1.PDF]

Supporting Information

1. Supplementary Data

Experimental section

***Preparation of Ni-MOF*:** $C_5H_8O_4$ ($HOOC(CH_2)_3COOH$, 0.3959 g), KOH (0.2292 g) and $Ni(CH_3COO)_2·4H_2O$ dissolved in a mixture solution (20 mL) containing 1:1 ethanol and deionized water. The mixture was then dispersed in a NaOH (2 mL, 0.4 M) aqueous solution under stirring. Subsequently, the solution was transferred into a Teflon-lined autocalve (50 mL) under the condition of 180 ^{o}C for 48 h, then cooled down to room temperature. Finally, the MOF was washed several times with deionized water and ethanol.

Preparation of Ni/NiO ultrathin nanobelts: To obtain Ni/NiO ultrathin nanobelts, the Ni-MIL-77 ultrathin nanobelts were heated in air at 350 ^{o}C with a temperature ramping rate of 1 ℃ min^{-1}.

Preparation of real samples: Firstly 12.5 mL borax saturated solution was added under a boiling water bath for 15 min, then 2.5 mL of 30% $ZnSO_4$ solution was used to precipitate protein. After cooled down, the resulting mixture was diluted to 50 mL.

Laviron's equation

$$E_{pa} = E^{0'} + 2.30\left(\frac{RT}{(1-\alpha)nF}\right)lgv$$

Where α is the electron transfer coefficient, n is the number of electron transferred, $E^{0'}$ is the formal potential, υ is the scan rate. R, T and F have their conventional meanings.

Section B. Supplementary Data

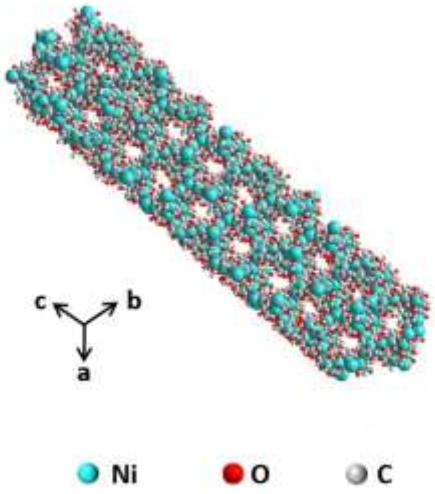

Figure S1. Structure diagram of Ni-MIL-77.

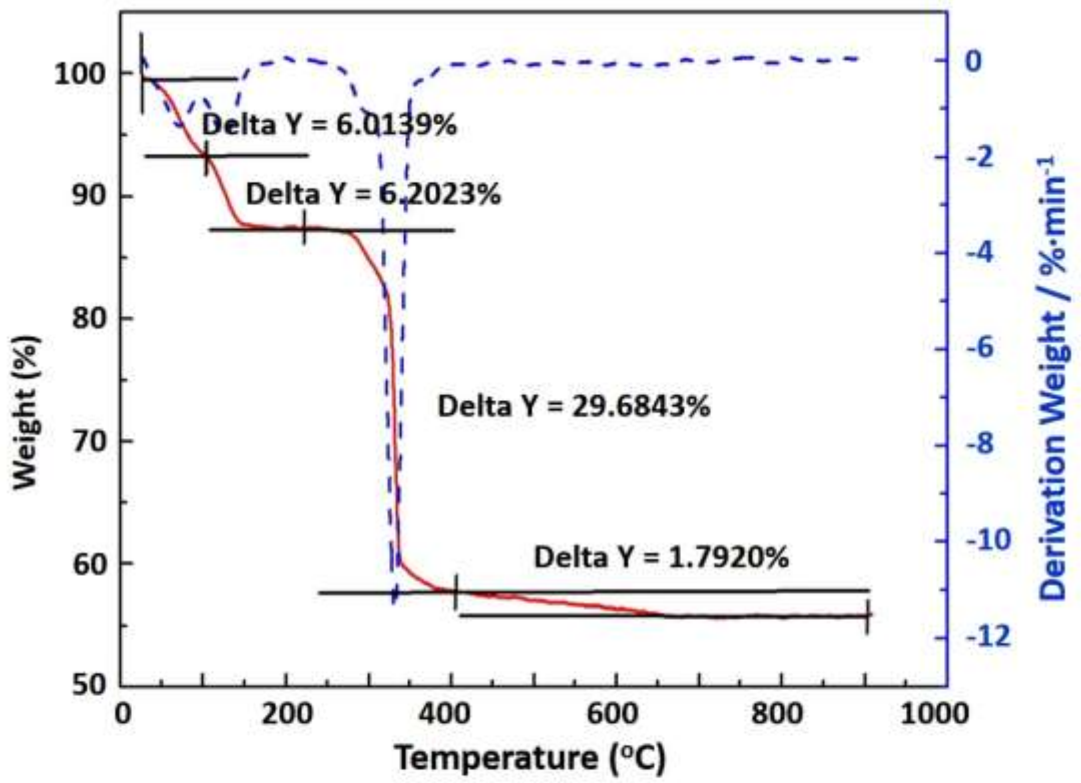

Figure S2. Thermogravimetric curve of Ni-MIL-77 in air with a heating rate of 1 ℃ min^{-1}.

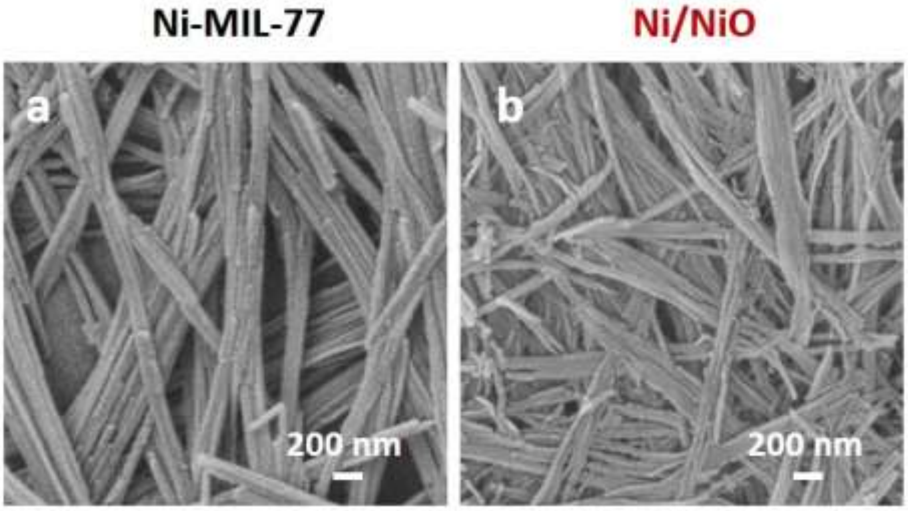

Figure S3. SEM images of a) Ni-MOF; b) Ni/NiO.

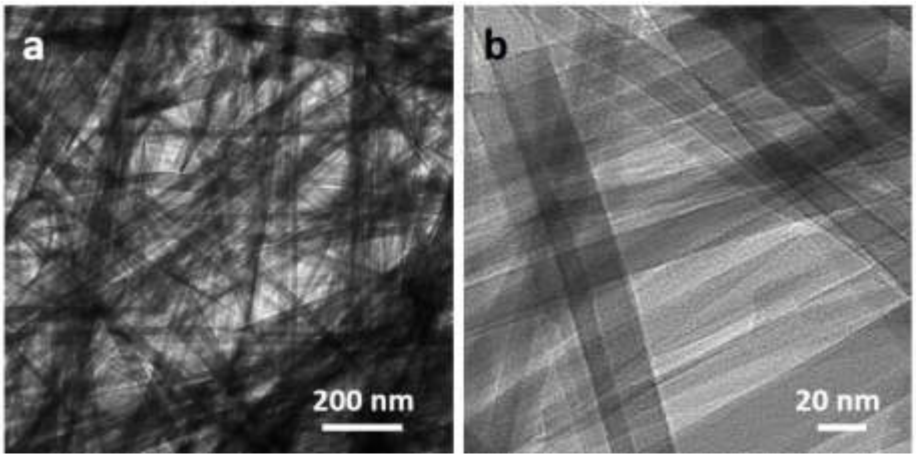

Figure S4. TEM images of Ni-MOF.

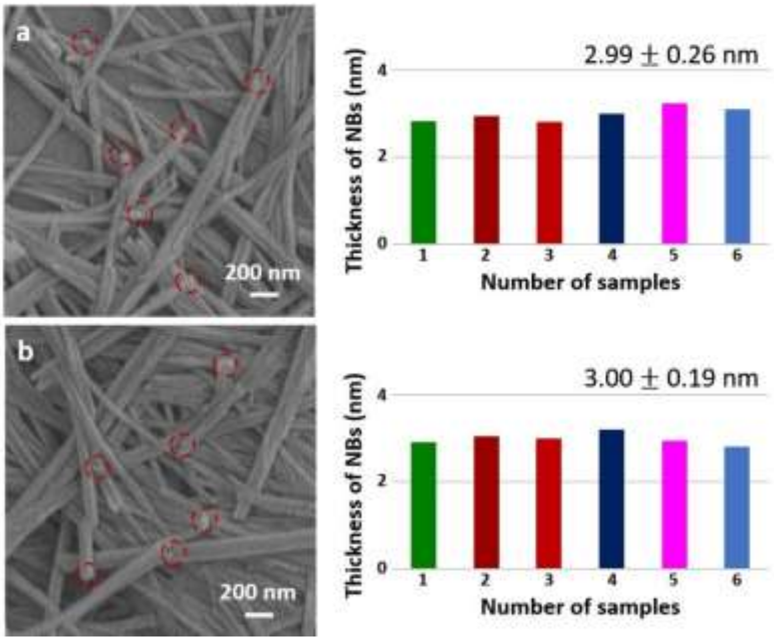

Figure S5. Analysis of the thickness of the selected area at random in the SEM images of Ni/NiO nanobelts, the right side is the corresponding size distribution histogram.

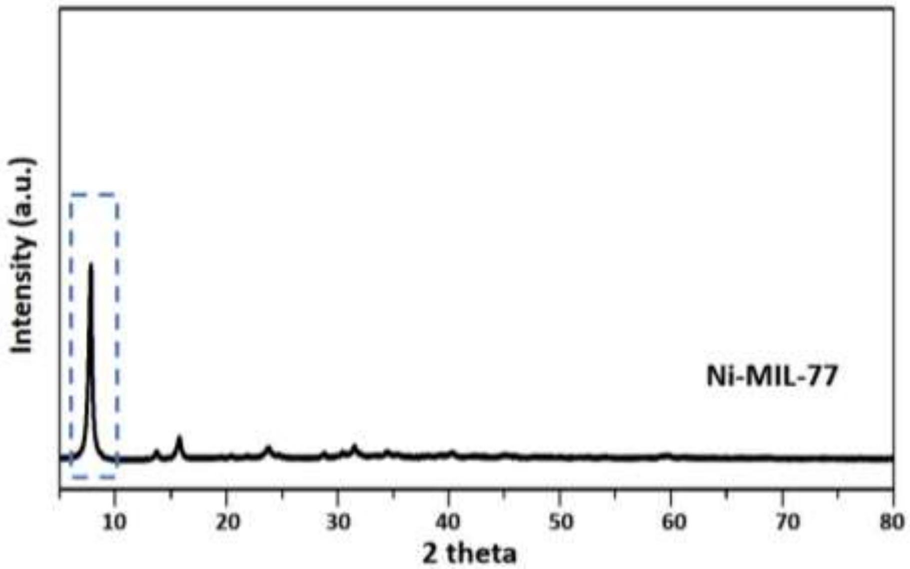

Figure S6. XRD patterns of the Ni-MIL-77, strong peak of 5º as the Ni-MIL-77 characteristic peak.(Guillou et al., 2003)

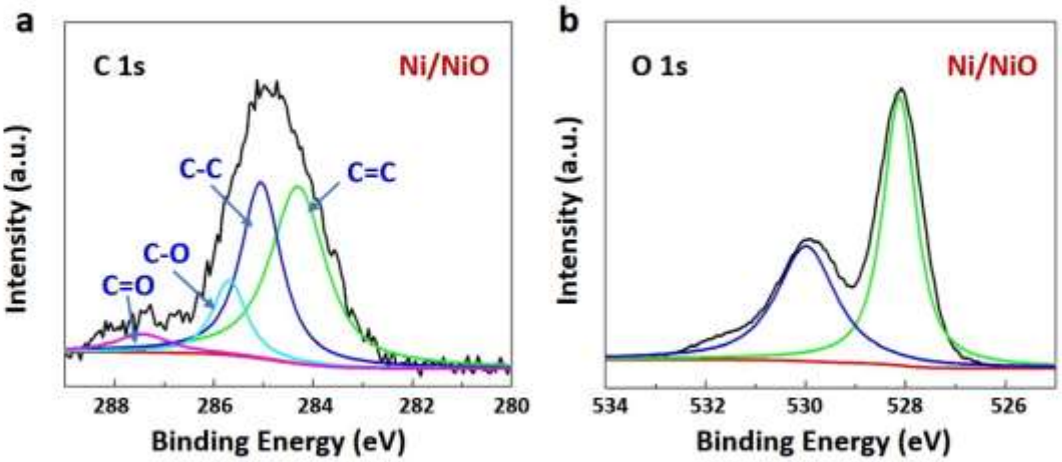

Figure S7. a) C 1s and b) O 1s XPS spectra of Ni/NiO.

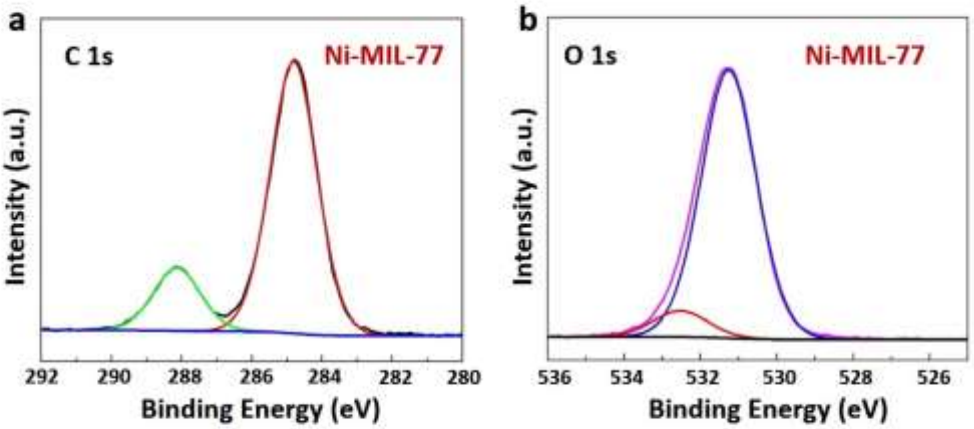

Figure S8. a) C 1s and b) O 1s XPS spectra of Ni-MIL-77.

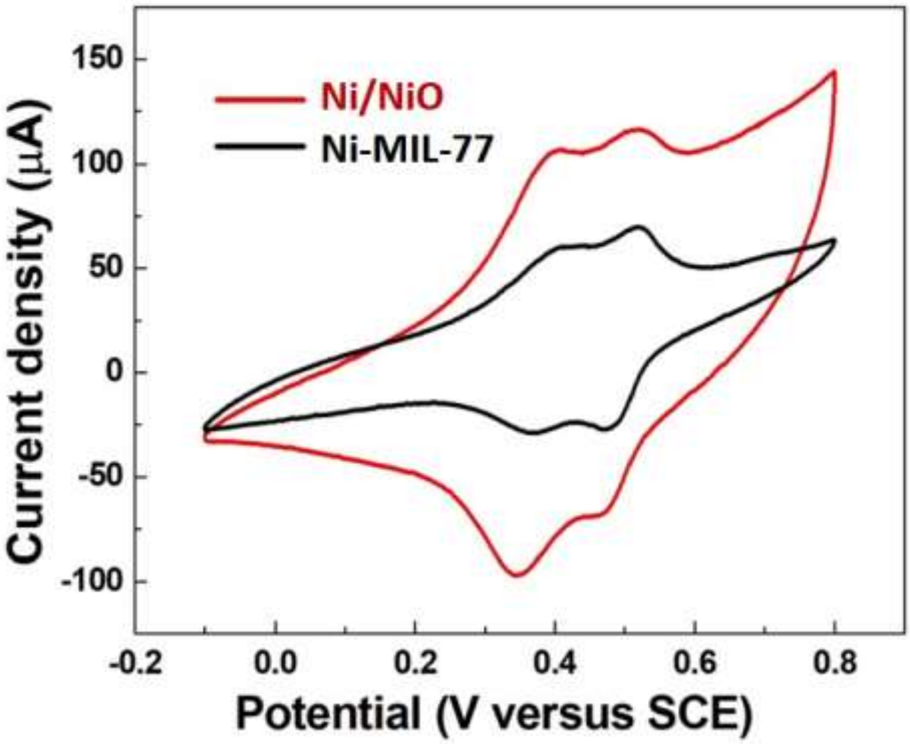

Figure S9. At a scan rate of 50 mV s^{-1} in 5.0 mM $K_3Fe(CN)_6$ + 1 M KCl solution, CV curves over a potential range of -0.1-0.8 V (versus SCE).

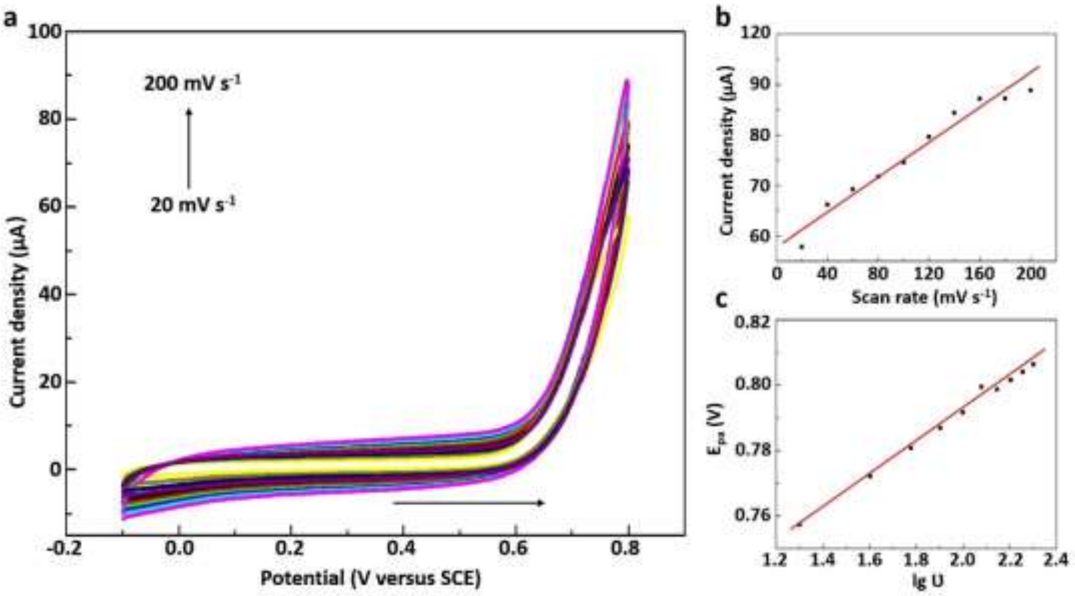

Figure S10. a) CVs of the Ni-MOF/GCE electrode in 0.1 M PBS (pH = 7.0) solution containing 5 mM $NaNO_2$ at scan rates from 20 to 200 mV s^{-1}. b) The plots of anodic peak currents to the scan rates. c) Anodic peak potentials versus lg υ. It can be found that the anodic peak currents increase linearly with the scan rate and the calibration equation is I_{pa} (μA) = 0.1695υ (mV s^{-1}) + 58.08382 (R^2 = 0.955).

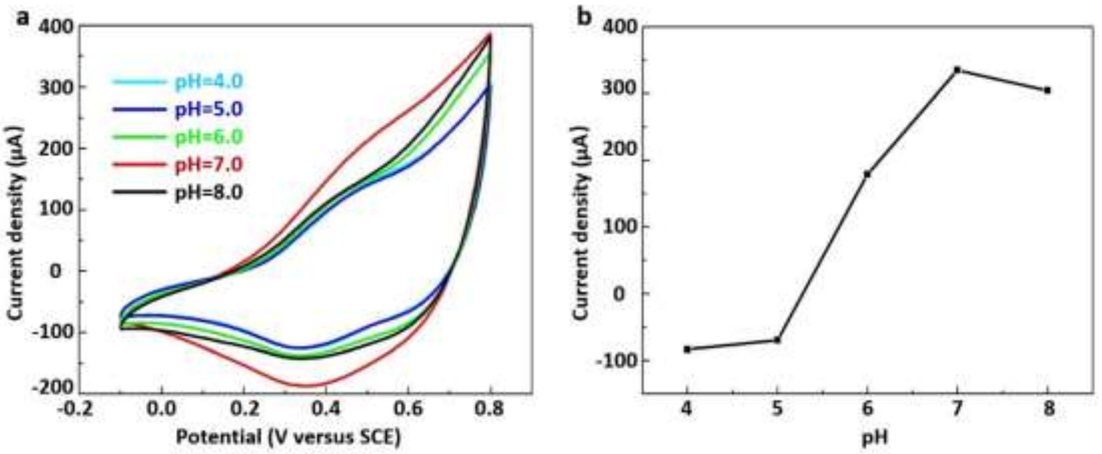

Figure S11. a) CVs of the Ni/NiO/GCE electrode in 0.1 M PBS solution containing 5 mM $NaNO_2$ with the pH ranging from 4.0 to 8.0, scan rate: 50 mV s^{-1}. b) The anodic peak current against pH.

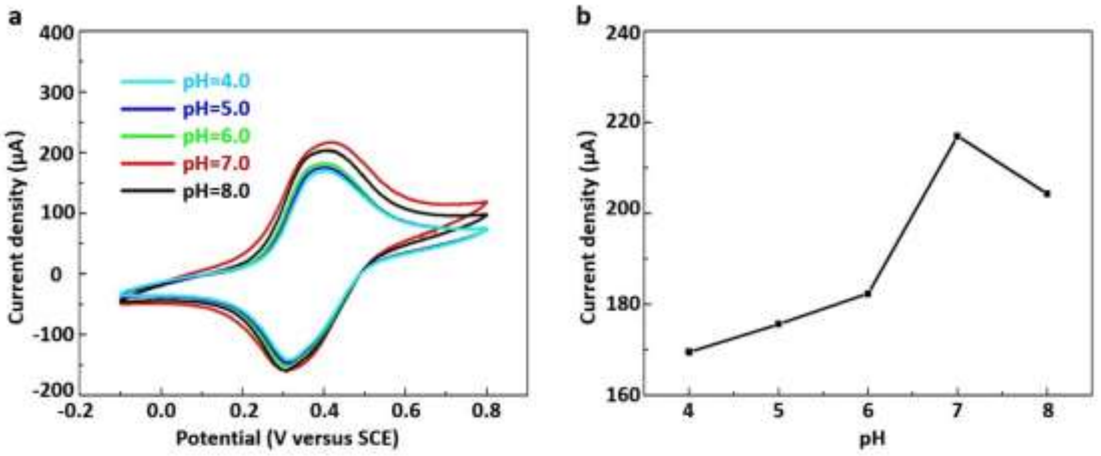

Figure S12. a) CVs of the Ni-MIL-77/GCE electrode in 0.1 M PBS solution containing 5 mM $NaNO_2$ with the pH ranging from 4.0 to 8.0, scan rate: 50 mV s^{-1}. b) The anodic peak current against pH.

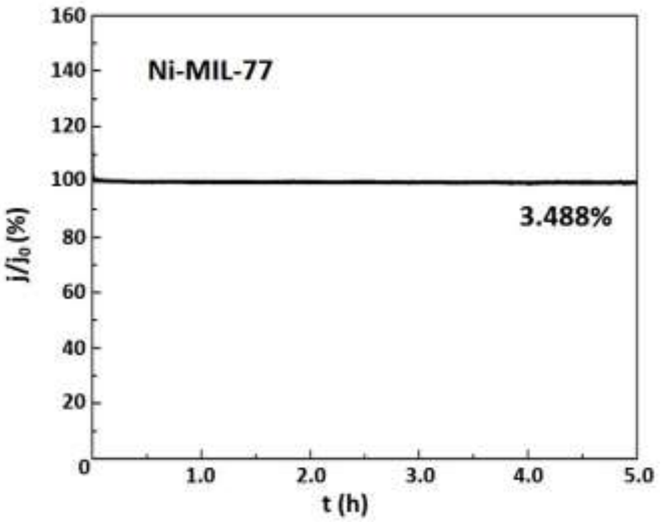

Figure S13. Stability of Ni-MOF/GCE in 0.1 M PBS (pH=7) with $NaNO_2$ over 5 h.

Table S1. Comparison of different measurements for $NaNO_2$ determination.

**Electrode**	**Limit of detection (μM)**	**Linear range (μM)**	**Sensitivity**	**Ref.**
CNSs	0.015	0.2-400	28.91 mA μM^{-1} cm^{-2}	(Yallappa et al., 2018)
AgNS	0.031	0.1-8	580 μA mM^{-1} cm^{-2}	(Shivakumar et al., 2017)
Co_3O_4/RGO	0.14	1-380	29.5 μA mM^{-1} cm^{-2}	(Haldorai et al., 2016)
NiO/MWCNTs/CP	0.25	1-100	3.53 μA μM^{-1} cm^{-2}	(Wan et al., 2017)
Fe_2O_3/rGO	0.015	0.05-780	0.204 μA μM^{-1} cm^{-2}	(Radhakrishnan et al., 2014)
$NiCo_2CO_3(OH)_2$	0.002	5-4000	1.21×10^{-4} μA mM^{-1} cm^{-2}	(Lu et al., 2017)
Fe_3O_4-rGO	0.3	0.5-9563	202.5 μA mM^{-1} cm^{-2}	(Bharath et al., 2015)
Pd/Fe_3O_4/polyDOPA/RGO	0.5	2.5-6470	0.01537 μA μM^{-1} cm^{-2}	(Zhao et al., 2017)
ERGO/AuNPs/SPCE	0.13	1-6000	0.3048 μA μM^{-1} cm^{-2}	(Jian et al., 2018)
CR-GO	1.0	8.9-167	0.0267 A M^{-1} cm^{-2}	(Mani et al., 2012)
$NiFe_2O_4$-CPE	0.1236	0.1-1000	7.9617 μA μM^{-1} cm^{-2}	(Nithyayini et al., 2019)
Cu-MOF/Au	0.082	0.1-4000	252 μA mM^{-1} cm^{-2}	(Chen et al., 2019)
MnO_2@g-C_3N_4	0.00123	0.01-1520	24.1777 μA μM^{-1} cm^{-2}	(Keerthi et al., 2019)
Co_3O_4-rGO/CNTs	0.016	0.1-8000	0.408 μA μM^{-1} cm^{-2}	(Zhao et al., 2019)
Ni/NiO NBs	0.25	0.5-1000	1.5319 μA mM^{-1} cm^{-2}	This work

Reference

Bharath, G., Madhu, R., Chen, S. M., Veeramani, V., Mangalaraj, D., and Ponpandian, N. (2015). Solvent-free mechanochemical synthesis of graphene oxide and Fe_3O_4-reduced graphene oxide nanocomposites for sensitive detection of nitrite. *J. Mater. Chem. A* 3, 15529-15539. doi:10.1039/c5ta03179f.

Chen, H., Yang, T., Liu, F., and Li, W. (2019). Electrodeposition of gold nanoparticles on Cu-based metal-organic framework for the electrochemical detection of nitrite. *Sensors Actuators, B Chem.* 286, 401-407. doi:10.1016/j.snb.2018.10.036.

Guillou, N., Livage, C., Drillon, M., and Férey, G. (2003). The Chirality, Porosity, and Ferromagnetism of a 3D Nickel Glutarate with Intersecting 20-Membered Ring Channels. *Angew. Chemie Int. Ed.* 42, 5314-5317. doi:10.1002/anie.200352520.

Haldorai, Y., Kim, J. Y., Vilian, A. T. E., Heo, N. S., Huh, Y. S., and Han, Y. K. (2016). An enzyme-free electrochemical sensor based on reduced graphene oxide/Co3O4 nanospindle composite for sensitive detection of nitrite. *Sensors Actuators, B Chem.* 227, 92-99. doi:10.1016/j.snb.2015.12.032.

Jian, J. M., Fu, L., Ji, J., Lin, L., Guo, X., and Ren, T. L. (2018). Electrochemically reduced graphene oxide/gold nanoparticles composite modified screen-printed carbon electrode for effective electrocatalytic analysis of nitrite in foods. *Sensors Actuators, B Chem.* 262, 125-136. doi:10.1016/j.snb.2018.01.164.

Keerthi, M., Manavalan, S., Chen, S.-M., and Shen, P.-W. (2019). A Facile

Hydrothermal Synthesis and Electrochemical Properties of Manganese dioxide@graphitic Carbon Nitride Nanocomposite toward Highly Sensitive Detection of Nitrite. *J. Electrochem. Soc.* 166, B1245-B1250. doi:10.1149/2.0251914jes.

Lu, S., Yang, C., and Nie, M. (2017). Hydrothermal synthesized urchin-like nickel-cobalt carbonate hollow spheres for sensitive amperometric detection of nitrite. *J. Alloys Compd.* 708, 780-786. doi:10.1016/j.jallcom.2017.03.059.

Mani, V., Periasamy, A. P., and Chen, S. M. (2012). Highly selective amperometric nitrite sensor based on chemically reduced graphene oxide modified electrode. *Electrochem. commun.* 17, 75-78. doi:10.1016/j.elecom.2012.02.009.

Nithyayini, K. N., Harish, M. N. K., and Nagashree, K. L. (2019). Electrochemical detection of nitrite at $NiFe_2O_4$ nanoparticles synthesised by solvent deficient method. *Electrochim. Acta* 317, 701-710. doi:10.1016/j.electacta.2019.06.026.

Radhakrishnan, S., Krishnamoorthy, K., Sekar, C., Wilson, J., and Kim, S. J. (2014). A highly sensitive electrochemical sensor for nitrite detection based on Fe_2O_3 nanoparticles decorated reduced graphene oxide nanosheets. *Appl. Catal. B Environ.* 148-149, 22-28. doi:10.1016/j.apcatb.2013.10.044.

Shivakumar, M., Nagashree, K. L., Manjappa, S., and Dharmaprakash, M. S. (2017). Electrochemical Detection of Nitrite Using Glassy Carbon Electrode Modified with Silver Nanospheres (AgNS) Obtained by Green Synthesis Using Pre-hydrolysed Liquor. *Electroanalysis* 29, 1434-1442. doi:10.1002/elan.201600775.

Wan, Y., Zheng, Y. F., Zhou, B., and Song, X. C. (2017). An Innovative

Electrochemical Sensor Ground on NiO Nanoparticles and Multi-Walled Carbon Nanotubes for Quantitative Determination of Nitrite. *J. Nanosci. Nanotechnol.* 18, 3585-3591. doi:10.1166/jnn.2018.14670.

Yallappa, S., Shivakumar, M., Nagashree, K. L., Dharmaprakash, M. S., Vinu, A., and Hegde, G. (2018). Electrochemical Determination of Nitrite Using Catalyst Free Mesoporous Carbon Nanoparticles from Bio Renewable Areca nut Seeds . *J. Electrochem. Soc.* 165, H614-H619. doi:10.1149/2.0561810jes.

Zhao, Z., Xia, Z., Liu, C., Huang, H., and Ye, W. (2017). Green synthesis of Pd/Fe_3O_4 composite based on polyDOPA functionalized reduced graphene oxide for electrochemical detection of nitrite in cured food. *Electrochim. Acta* 256, 146-154. doi:10.1016/j.electacta.2017.09.185.

Zhao, Z., Zhang, J., Wang, W., Sun, Y., Li, P., Hu, J., et al. (2019). Synthesis and electrochemical properties of Co_3O_4-rGO/CNTs composites towards highly sensitive nitrite detection. *Appl. Surf. Sci.* 485, 274-282. doi:10.1016/j.apsusc.2019.04.202.
